# Afferent Visual Pathway Affection in Patients with PMP22 Deletion-Related Hereditary Neuropathy with Liability to Pressure Palsies

**DOI:** 10.1371/journal.pone.0164617

**Published:** 2016-10-17

**Authors:** Alexander U. Brandt, Elena Meinert-Bohn, Jan Leo Rinnenthal, Hanna Zimmermann, Janine Mikolajczak, Timm Oberwahrenbrock, Sebastian Papazoglou, Caspar F. Pfüller, Johann Schinzel, Björn Tackenberg, Friedemann Paul, Katrin Hahn, Judith Bellmann-Strobl

**Affiliations:** 1 NeuroCure Clinical Research Center, Charité –Universitätsmedizin Berlin, Berlin, Germany; 2 Institute of Neuropathology, Charité –Universitätsmedizin Berlin, Berlin, Germany; 3 Department of Neurology, University of Giessen-Marburg, Marburg, Germany; 4 Department of Neurology, Charité –Universitätsmedizin Berlin, Berlin, Germany; 5 Experimental and Clinical Research Center, Max Delbrueck Center for Molecular Medicine and Charité –Universitätsmedizin Berlin, Berlin, Germany; Universidad de Jaen, SPAIN

## Abstract

**Background:**

The PMP22 gene encodes a protein integral to peripheral myelin. Its deletion leads to hereditary neuropathy with liability to pressure palsies (HNPP). PMP22 is not expressed in the adult central nervous system, but previous studies suggest a role in CNS myelin development. The objective of this study was to identify potential structural and functional alterations in the afferent visual system in HNPP patients.

**Methods:**

Twenty HNPP patients and 18 matched healthy controls (HC) were recruited in a cross-sectional study. Participants underwent neurological examination including visual acuity, visual evoked potential (VEP) examination, optical coherence tomography (OCT), and magnetic resonance imaging with calculation of brain atrophy, regarding grey and white matter, and voxel based morphometry (VBM), in addition answered the National Eye Institute’s 39-item Visual Functioning Questionnaire (NEI-VFQ). Thirteen patients and 6 HC were additionally examined with magnetic resonance spectroscopy (MRS).

**Results:**

All patients had normal visual acuity, but reported reduced peripheral vision in comparison to HC in the NEI-VFQ (p = 0.036). VEP latency was prolonged in patients (P_100_ = 103.7±5.7 ms) in comparison to healthy subjects (P_100_ = 99.7±4.2 ms, p = 0.007). In OCT, peripapillary retinal nerve fiber layer thickness RNFL was decreased in the nasal sector (90.0±15.5 vs. 101.8±16.5, p = 0.013), and lower nasal sector RNFL correlated with prolonged VEP latency (Rho = -0.405, p = 0.012). MRS revealed reduced tNAA (731.4±45.4 vs. 814.9±62.1, p = 0.017) and tCr (373.8±22.2 vs. 418.7±31.1, p = 0.002) in the visual cortex in patients vs. HC. Whole brain volume, grey and white matter volume, VBM and metabolites in a MRS sensory cortex control voxel did not differ significantly between patients and HC.

**Conclusion:**

PMP22 deletion leads to functional, metabolic and macro-structural alterations in the afferent visual system of HNPP patients. Our data suggest a functional relevance of these changes for peripheral vision, which warrants further investigation and confirmation.

## Introduction

The PMP22 gene on chromosome 17p11.2 encodes a 22-kD protein compound of myelinated fibers in the peripheral nervous system [1]. PMP22 protein’s precise function is unknown. Studies in PMP22+/- mice indicate that PMP22 haploinsufficiency predominantly affects myelin junction formation or maintenance in peripheral Schwann cells, thereby impairing nerve conduction [2,3]. Mutations in the PMP22 gene are the underlying cause of several hereditary neuropathies (for a recent review see [4]): Gene *duplication* causes Charcot-Marie-Tooth disease type 1A (CMT1A) ([5,6]. Gene *deletion* leads to hereditary neuropathy with liability to pressure palsies (HNPP) [7]. *Point mutations* can cause neuropathies with mixed phenotypes ranging between CMT1A and HNPP [8].

HNPP is an episodic, multifocal peripheral neuropathy with typically recurrent transient pressure palsies without pain. The focal motor and sensory symptoms usually occur within single nerves [9]. Reports of regional HNPP prevalence range between 7.2 cases per 100,000 in Northern England [10] and 16 per 100,000 in Finland [11].

In the central nervous system (CNS), PMP22 mRNA was initially detected during development of the neural crest [12,13]. mRNA and protein were identified in the cytoplasm of CNS neurons [14] and in oligodendrocytes in low concentrations [15]. In oligodendrocytes, PMP22 is thought to be relevant for myelin regulation, and not to be a structural component of myelin [15].

That PMP22 deletion also plays a pathologic role in the CNS is supported by several studies that found CNS involvement or white matter lesions in patients with HNPP [16–20]. Recently, a study with 15 HNPP patients reported decreased white matter brain volume, suggesting widespread alterations in the CNS of HNPP patients [21]. Moreover, a specific affection of the visual system has been reported in a HNPP family [22] and in a study comprising 8 HNPP patients [23]. In both studies, a mild latency prolongation of the P100 wave in visually evoked potentials (VEP) examinations was present in some patients.

Against these findings, the aim of our study was to comprehensively investigate a potential involvement of the visual pathway in HNPP using a multimodal imaging and electrophysiology approach comprising VEP, optical coherence tomography (OCT) [24] and brain magnetic resonance imaging (MRI), including magnetic resonance spectroscopy (MRS) in combination with visual functional data (NEI-VFQ).

## Material and Methods

### Patients and Controls

For this cross-sectional study subjects were recruited from the Charité –Universitätsmedizin Berlin’s neuroimmunology outpatient clinic and from healthy volunteers. A total of 20 genetically confirmed HNPP patients with PMP22 deletion of chromosome 17p11.2–12 (13 female, 7 male) and 18 age- and sex-matched healthy controls (HC) were examined. One HC was excluded due to papilledema, leaving a total of 17 HC (11 female, 6 male). Sex distribution did not differ significantly between the groups (p = 0.985). Age of HNPP patients (41±15 years) and HC (39±15 years) was not statistically different (p = 0.784). Exclusion criteria for patients and controls were any other neurological, ophthalmological or other disease potentially causing retinal abnormalities, i.e. glaucoma and diabetes mellitus. All patients received a neurological examination including testing for high contrast visual acuity using Snellen charts.

The study was approved by the ethics committee of the Charité –Universitätsmedizin Berlin and was conducted in accordance with the currently applicable version of the Declaration of Helsinki. All participants gave written informed consent.

### Vision-related quality of life

Vision-related quality of life was assessed by self-administered questionnaire using the validated German 39-item version of the U.S. National Eye Institute Visual Functioning Questionnaire (NEI-VFQ39)[25] as previously described in detail [26]. The NEI-VFQ computes 12 subscores relating to different aspects of vision-related daily activities and an overall composite score. Each score can range from 0 to 100, with a value of 100 representing the best possible score, and a value of 0 the worst possible score for complete loss of vision-related function.

### Optical Coherence Tomography

Peripapillary retinal nerve fiber layer (pRNFL) thickness and macular volume scans were acquired using Heidelberg Spectralis spectral-domain OCT (Heidelberg Engineering, Germany). pRNFL thickness was determined from a 12° circular scan with approximately 3.4 mm diameter around the optic nerve head using the device’s standard protocol with activated eye tracker. Whenever possible, the maximum 100 averaging frames in the automatic-real-time mode (ART) were used. Average and papulomacular (PMB) pRNFL were directly derived from the device’s output. pRNFL thickness of the nasal sector was calculated from the device’s output of the nasal (N), nasal-superior (NS) and nasal-inferior (NI) sectors using the formula: Nasal pRNFL = (N+2xNS+2xNI)/5, accounting for the twice-as-big area covered by the N sector in comparison to the NS and NI sectors. Macular volume was measured using a custom protocol that generated 61 vertical slices (B-scans) focusing on the fovea, at scanning angle of 30°×25° and a resolution of 768 A-scans per B-scan and ART 13. Total macular volume (TMV) was calculated by estimating the distance between the inner limiting membrane and Bruch´s membrane in a 6 mm-diameter cylinder using the OCT software's standard segmentation algorithm. All scans were acquired by experienced operators and were evaluated for sufficient signal strength, correct centering and segmentation based on the OSCAR-IB criteria by a second operator [27].

Intraretinal segmentation data were taken from a manufacturer’s beta-software (Heidelberg Eye Explorer V1.8.6.0 with Spectralis Viewing Module V6.0.0.2). This software automatically detects boundaries between retinal layers. All automated segmentation results were manually inspected and corrected if necessary by a single experienced grader. Combined ganglion cell and inner plexiform layer (GCIP) and the inner nuclear layer (INL) were analyzed as volume within the standard 6 mm ETDRS ring around the fovea as previously described [28]. Intraretinal segmentation could not be performed for three eyes from three patients, because of low image quality. These scans were excluded from intraretinal layer analysis but included in the pRNFL and TMV analysis.

### Visually Evoked Potentials

The Dantec^TM^ Keypoint VEP system (Natus Europe GmbH, Planegg, Germany) was used to present a single-eye full field, black-and-white checkerboard pattern (15’/50-60’, at 1 m). Electrodes were placed on Oz-Fz according to the “10–20 International System”. We recorded the 500 ms period following each visual stimulus, induced an average of 100 times for each eye and checked for reproducibility using a second run. We used P_100_ latency for analysis only; P_100_ amplitude was not analyzed. Abnormal values were defined as the mean value + 2 standard deviations in accordance with our clinical practice (above 111 ms). VEP were measured prior to or on the same day as OCT.

### Magnetic Resonance Imaging

MRI was performed on 16 HC and 18 HNPP patients. Subjects received a high-resolution whole-brain scan at the Imaging Science Institute Charité-Siemens (ISI, Berlin, Germany) on a 1.5T MRI scanner (Magnetom Avanto, Siemens, Erlangen, Germany) using a T1-w magnetization-prepared rapid gradient echo (MPRAGE) sequence. MRI parameters were: TR = 1,900 ms, TE = 3.09 ms, TI = 1,100 ms, flip angle = 15°, direction of slice selection left -> right (sagittal slicing), matrix size = 160x480x512 voxel size = 1x0.5x0.5 mm.

### Brain Atrophy and Voxel Based Morphometry

Brain atrophy was assessed as normalized brain volume (NBV), normalized grey matter volume (NGMV) and normalized white matter volume (NWMV) on basis of the T1-w scans using the FSL 5.0 (FMRIB Software Library, Oxford, UK) pipeline SIENAX [29]. Prior to SIENAX, each scan was cropped to an area approximating the MNI standard space template shipped with FSL and subsequently corrected for non-uniformity using the N3 algorithm from MIPAV version 5.4.2 [30].

Voxel-based morphometry was performed using VBM12 (University of Jena, Jena, Germany) for SPM12 (Wellcome Trust Centre for Neuroimaging, London, UK). Prior to submitting the T1w images to the VBM12 pipeline, the images were cropped to an MNI space area and reoriented to ensure approximate coincidence of the anterior commissure with the origin of coordinates. A linear model was built for voxel-wise testing for group differences in local grey matter density between HNPP patients and healthy controls. The linear model comprised age, gender and total intracranial volume as covariates of no interest in order to mitigate any possible skewing of results. Significance was determined by family-wise error corrected results at p = 0.05.

### Magnetic Resonance Spectroscopy

**Fig 1:**
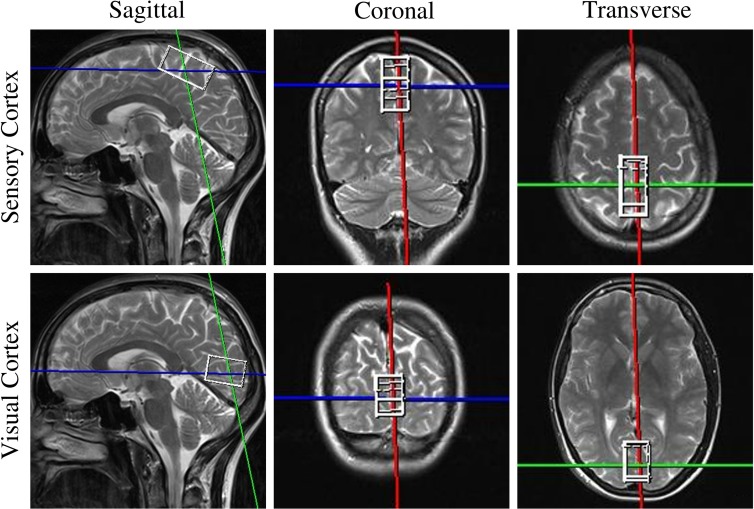
MRS voxel placement. Representative voxel placement in magnetic resonance spectroscopy in the sensory cortex (upper row) and visual cortex (lower row). Both voxels are mixed tissue voxels comprising both grey and white matter.

MRS was performed on 6 HC and 13 HNPP patients. All spectra were acquired in a single session with MRI with a standard circular polarized head coil. Sagittal, coronal and transversal T2-w turbo-spin-echo MR images were acquired for voxel positioning. Single-voxel 1H-spectra were obtained using the point-resolved spectroscopy sequence (PRESS) [31] with frequency-selective water suppression (CHESS) [32] and with the suppression-bandwidth set to 50 Hz. An echo-time of TE = 135 ms was used to extract the signals from NAA, Cr and Cho resonances. For this reason, the fit-quality for these metabolites was optimized. The repetition time was set to TR = 3,000 ms in order to increase the signal-to-noise-ratio and to reduce interfering T1 contrast. After 4 pre-scans, 192 acquisitions were added, resulting in an acquisition time of 9.8 min for each spectrum. Before acquisition, local shimming was performed using the unsuppressed water signal, followed by adjustment of the frequency-selective pulses for water suppression. A voxel size of 40x20x20 mm for sensory cortex (SC) and 30x20x20 mm for visual cortex (VC) measurements delivered excellent spectral quality (Fig 1). Voxels were precisely positioned along the mid-sagittal plane in order to minimize white matter partial volume effects. Spectrum analysis was performed using the LCModel software package with the standard basis set for 1.5 T and TE = 135 ms [33].

### Statistical Analysis

Demographic group differences were analyzed using Student’s t-test (for age) and Pearson’s Chi^2^ test (for gender). NEI-VFQ group differences were analyzed using non-parametric Mann-Whitney-U tests. Generalized estimating equation models (GEE) were used with the working correlation matrix “Exchangeable” for comparison of OCT and VEP data between patients and controls taking within-patient inter-eye dependencies in account. In each case, OCT/VEP measurement values were used as dependent and the parameter ‘group’ as independent variables. General linear models (GLM) were used to compare the MRI and MRS measurements of groups. All GEE and GLM were corrected for sex and age as covariates. Correlation analysis between OCT and VEP parameters was performed using non-parametric Spearman’s Rho analyses. Statistical analyses were performed with SPSS version 22 (IBM SPSS, USA). Statistical significance was achieved at p < 0.05. Measurement parameters are given in mean ± standard deviation unless otherwise noted. No previous sample size calculation was performed. The reader should be aware that results from this study are exploratory and all p values (except VBM) are uncorrected for multiple comparisons.

## Results

All patients had normal visual acuity and did not report any visual symptoms in the medical examination. However, in the NEI-VFQ questionnaire asking for several aspects of vision-related quality of life, patients scored significantly lower in the peripheral vision category than HC, whereas all other areas were similar to HC (Table 1).

**Table 1:** Vision-related quality of life.

	HNPP				HC				MWU
	Mean	SD	Min	Max	Mean	SD	Min	Max	p
NEI-VFQ39 Composite Score	88	9	72	100	92	8	68	100	0.129
General Health*	*53*	*21*	*0*	*83*	*76*	*14*	*43*	*95*	***0*.*001***
General Vision	72	15	50	95	70	16	45	95	0.856
Ocular Pain	81	17	50	100	89	14	50	100	0.133
Near Vision	89	10	63	100	91	15	46	100	0.122
Distance Vision	90	9	71	100	95	7	71	100	0.109
Social Functions	96	8	75	100	98	5	83	100	0.612
Well Being & Distress	89	11	70	100	93	10	60	100	0.214
Role Limitations	83	20	31	100	91	14	63	100	0.210
Dependency	99	3	88	100	100	2	94	100	0.704
Driving	80	17	50	100	90	13	58	100	0.100
Color Vision	98	8	75	100	98	6	75	100	0.899
Peripheral Vision	86	17	50	100	98	6	75	100	**0.036**

Abbreviations: NEI-VFQ39, 39-item National Eye Institute Visual Functioning Questionnaire; HC, healthy controls; MWU, Mann-Whitney U test; SD, standard deviation.

*) The general health score asks for general non-vision-related health and is not included in the NEI-VFQ composite score. Significant p-values in bold.

**Fig 2:**
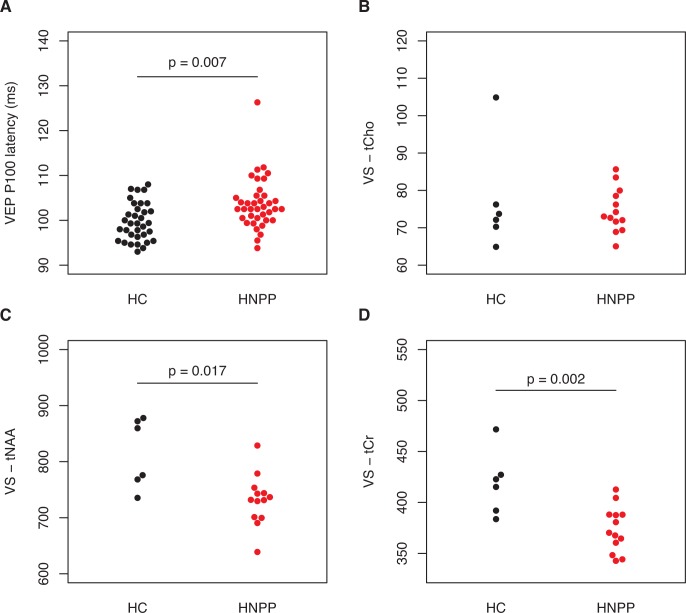
VEP and MRS. Differences in visual evoked potential (VEP) measurements and magnetic resonance spectroscopy between HNPP patients (in red) and healthy controls (HC, in grey). A) VEP P100 latency, B) visual cortex tCho, C) visual cortex tNAA, D) visual cortex tCr. P values are derived from multiple linear regression models (see results).

VEP latency was significantly prolonged in HNPP patients (P_100_ = 103.7±5.7 ms) in comparison to HC (P_100_ = 99.7±4.2 ms, GEE B = 4.0, SE = 1.5, p = 0.007) (Fig 2A). However, compared to normative data only one eye from one patient was out of normal range (P_100_ = 123 ms). Four eyes from three patients were borderline normal (P_100_ > = 111 ms).

**Fig 3:**
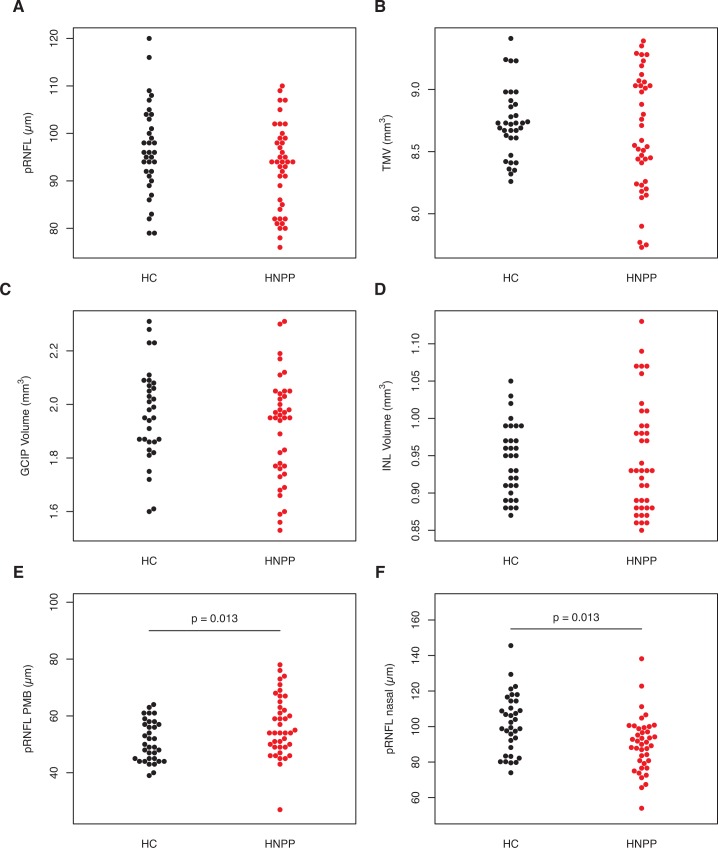
Optical Coherence Tomography. Differences in retinal optical coherence tomography measurements between HNPP patients (in red) and healthy controls (HC, in grey). A) Peripapillary retinal nerve fiber layer thickness (pRNFL), B) pRNFL in the papulomacular bundle (PMB), C) pRNFL in the nasal hemisphere, D) total macular volume (TMV), E) ganglion cell and inner plexiform layer (GCIP), F) inner nuclear layer (INL). P values are derived from generalized estimating equation models.

Average pRNFL, TMV, GCIP and INL were not significantly different between patients and healthy controls (Table 2 and Fig 3). However, to further investigate a potential selective affection of peripheral vision suggested from the vision-related quality of life questionnaire we analyzed the pRNFL sections of the papulomacular bundle, which contains primarily central vision axons, and the nasal hemisphere, which primarily contains axons from the peripheral retina. Here, pRNFL in the PMB was significantly increased in HNPP vs. HC, whereas pRNFL in the nasal hemisphere was significantly reduced in HNPP patients, supporting the peripheral vision affection (Table 2). Furthermore, reduced nasal pRNFL thickness was inversely correlated with increased VEP P100 latency (Spearman’s Rho = -0.405, p = 0.012), whereas PMB pRNFL thickness was not (p = 0.499). Noteworthy, two female patients, 41 and 71 years old, showed bilateral below normal TMV and pRNFL values. The 71-year-old patient presented with focal vitreous traction related damage in one eye, but no further pathologies were apparent that could potentially explain these abnormal values. VEP latency was normal in these eyes (99.4–104.0 ms).

**Table 2:** OCT measurements.

	HNPP				HC				GEE		
	Mean	SD	Min	Max	Mean	SD	Min	Max	B	SE	p
**pRNFL (μm)**	92.9	9.1	76.0	110.0	96.5	10.0	79.0	120.0	-3.4	2.9	0.237
**PMB RNFL (μm)**	56.5.	10.7	27.0	78.0	51.1	7.1	39.0	64.0	5.5	2.2	0.013
**Nasal RNFL (μm)**	90.0	15.5	54.0	138.2	101.8	16.5	74.0	145.6	-11.7	4.7	0.013
**TMV (mm³)**	8.65	0.48	7.73	9.39	8.73	0.28	8.26	9.41	-0.08	0.12	0.507
**GCIP (mm³)**	1.91	0.2	1.53	2.31	1.96	0.17	1.6	2.31	-0.06	0.05	0.246
**INL (mm³)**	0.95	0.08	0.85	1.13	0.94	0.05	0.87	1.05	<0.01	0.02	0.827

Abbreviations: HC, healthy controls; GEE, generalized estimating equation models; SD, standard deviation; B, non-standardized coefficient from GEE; SE, standard error; pRNFL, peripapillary retinal nerve fibre layer thickness; PMB, papulomacular bundle; TMV, total macular volume; GCIP, ganglion cell and inner plexiform layer; INL, inner nuclear layer.

Normalized brain, grey matter and white matter volumes were not significantly different between patients and healthy controls (Table 3). Voxel based morphometry did not reveal any regional grey matter volume differences between patients and HC (all p > 0.05, not shown).

**Table 3:** Brain atrophy.

	HNPP				HC				MLR	
	Mean	SD	Min	Max	Mean	SD	Min	Max	F	p
**Brain atrophy**										
NBV (ml)	1,558	69	1,438	1,679	1,546	77	1,423	1,682	3.488	0.081
NGMV (ml)	783	57	706	929	772	62	680	901	1.268	0.278
NWMV (ml)	775	30	718	826	774	30	718	840	2.307	0.150

Abbreviations: HC, healthy controls; MLR, multiple linear regression; SD, standard deviation; NBV, normalized brain volume; NGMV, normalized grey matter volume; NWMV, normalized white matter volume.

MRS was only available for a subset of participants (6 HC and 13 HNPP patients), who were similar in age (p = 0.172) and gender (p = 0.622). In the visual cortex voxel, MRS, tNAA and tCr were significantly reduced in patients (Table 3 and Fig 2B–2D). When normalizing metabolites against tCr, tCho/tCr was increased in patients (0.200 vs. 0.183) but this difference did not reach statistical significance (p = 0.094), probably due to error propagation, because normalization to tCr introduces another error into the calculation, namely, the error of the tCr measurement. Visual cortex tNAA (GEE B < 0.001, SE = 0.0, p = 0.266) or tCr (GEE B = 0.0, SE = 0.0, p = 0.919) did not correlate with VEP latency in patients. MRS metabolites in the sensory cortex control voxel did not differ between HNPP and controls (Table 4).

**Table 4:** MRS measurements.

	HNPP				HC				MLR	
	Mean	SD	Min	Max	Mean	SD	Min	Max	F	p
**Visual cortex**										
tCho	74.7	5.9	65.0	85.6	77.0	14.2	64.9	104.9	0.248	0.626
tNAA	731.4	45.4	638.9	828.7	814.9	62.1	735.4	877.9	7.179	**0.017**
tCr	373.8	22.2	342.7	412.7	418.7	31.1	383.6	471.7	13.766	**0.002**
**Sensory cortex**										
tCho	76.7	12.6	51.3	103.8	79.0	16.4	61.4	103.9	0.102	0.754
tNAA	584.8	77.2	442.7	699.6	621.7	52.0	549.6	698.8	0.017	0.898
tCr	315.8	37.8	236.7	380.8	339.6	29.9	305.2	378.9	0.335	0.572

Abbreviations: MRS, magnetic resonance spectroscopy; HC, healthy controls; MLR, multiple linear regression; SD, standard deviation.

## Discussion

We here report functional and neurochemical involvement of the afferent visual system in HNPP patients. Despite patients having visual acuity within normal range, patients reported problems with peripheral vision in comparison to HC. VEP latencies were prolonged in patients in comparison to healthy subjects. Average retinal OCT layer thicknesses in patients did not differ from healthy subjects, but nasal pRNFL thickness, where most of the axons from the peripheral retina run, was significantly reduced. Furthermore, reduced nasal pRNFL thickness was associated with prolonged VEP latencies. MRS in a subset of participants revealed reduced tNAA and tCr in a visual cortex voxel but not in a sensory cortex control voxel in patients. Whole brain volume, grey and white matter volumes, regional grey matter volumes and metabolites in a sensory cortex voxel were also comparable to healthy controls.

VEP alterations have been previously reported in a smaller study comprising 8 HNPP patients [23]. Our study confirms these findings. Interestingly, in our patients this was associated with a mild decrease of RNFL thickness in the nasal sectors, and a mild increase of temporal RNFL in the papulomacular bundle. In normal healthy eyes, foveal or macular axons mainly form the temporal RNFL, whereas axons from ganglion cells in the peripheral retina run through superior, inferior and especially nasal sectors [34]. The observed VEP and OCT alterations potentially reflect ultra-structural or functional myelin changes. Notably, conduction slowing in HNPP has been also shown for other cranial nerves, including the acoustic, trigeminal and facial nerves [23,35].This is supported by studies in PMP22 heterozygous mice, in which PMP22 haplodeficiency leads to conduction slowing in peripheral nerves, pointing towards potentially similar mechanisms in CNS myelin [2,3]. In the CNS, PMP22 is a potential factor involved in cellular growth regulation during early development [13,12,36]. Accordingly, early disturbances of cranial nerves have been described and proposed as the cause of symptoms like deafness in some patients with PMP22 mutations [37].

In contrast, our findings argue against a potential damage-related mechanism. Symptoms in HNPP patients are regularly induced from damage to peripheral nerves, i.e. due to pressure on the resp. nerves. Damage to CNS myelin by a similar mechanism seems unlikely given the protective localization of the CNS. However, they are conceivable in the optic nerve, which is especially susceptible to stress at its exit from the eye, where it is additionally subject to eye movements and potentially movement-induced stress. However, in this case one would have expected pan or temporal retinal atrophy from retrograde neurodegeneration, which was clearly not the case in our study.

Apart from the VEP results, HNPP patients also showed neurochemical alterations in the visual cortex. We used an optimized MRS protocol for yielding high-quality concentrations of tNAA, tCho and tCr. Interestingly, tNAA and tCr were significantly reduced in HNPP patients whereas tCho was not. N-Acetylaspartic acid (NAA) and N-acetylaspartylglutamic acid (NAAG) are predominantly localized in neurons [38], which is why tNAA (NAA+NAAG) is considered a neuronal marker. Therefore, loss of tNAA is generally interpreted as neuronal damage or neuronal loss. In contrast, tCho is generally considered a marker for cell membrane turnover [39]. tCr, on the other hand, is often used as a housekeeping metabolite to such an extent that some studies normalize other metabolites against tCr concentration [40]. When normalizing against tCr, tCho/tCr was elevated in comparison to healthy controls, although this difference did not reach significance. Taken together, the tNAA and tCr reduction and a trend towards relative tCho increase suggest an altered visual cortex white and grey matter composition, e.g. during development, rather than signs of neurodegeneration. Our MRS findings therefore potentially support a role of PMP22 in CNS white matter development. This is in line with a recent study that did not find any PMP22 expression in the adult CNS, but hypomyelination-sparing U fibers in brain white matter [21]. It is therefore conceivable that we detected these previously described findings from biopsies with MRS in our study. This is supported by a recent report about fractional anisotropy abnormalities in central nervous system white matter detected with diffusion tensor imaging in HNPP patients.[41] Alternatively, the detected MRS alterations might reflect functional changes resulting from altered nerve conduction in HNPP patients rather than structural changes.

In contrast to Chanson et al., our patients had white matter volume measurements in the range of healthy controls [21]. In fact, we did not find any evidence for reduced global or regional brain volumes in our patients using SIENAX and VBM analysis.

The most important limitation of our study is the small sample size, owing to the rarity of the disease. Additionally, MRS was only available for a subset of participants. In MRS, both voxels were mixed tissue voxels, and this voxel composition might have been inappropriate to reflect white matter changes in the sensory voxel adequately. It is therefore possible that similar white matter changes are also present in the sensory cortex white matter voxel, but the mixed voxel positioning did not include enough white matter tissue to reflect this. Importantly, we only assessed high contrast visual acuity and subjective vision-related quality of life in this study; methods that are able to detect subtle changes in clinical visual function should be implemented in a future study. To confirm a structural and functional alteration of the optic nerve with functional relevance for peripheral vision, future studies should include visual fields and multifocal VEP. Lastly, the reader should be aware that this was an exploratory study and our findings could be skewed by the small sample size. Independent confirmation is warranted.

In summary, we show evidence that PMP22 deletion leads to functional and metabolic alterations in the CNS of HNPP patients. Taken together, our results and those of previous studies suggest that CNS white matter, but not grey matter is altered in adult patients, even if PMP22 is not expressed in the adult CNS.
